# Evaluation of Cepheid's Xpert MTB/RIF Test on Pleural Fluid in the Diagnosis of Pleural Tuberculosis in a High Prevalence HIV/TB Setting

**DOI:** 10.1371/journal.pone.0102702

**Published:** 2014-07-22

**Authors:** John K. Lusiba, Lydia Nakiyingi, Bruce J. Kirenga, Agnes Kiragga, Robert Lukande, Maria Nsereko, Willy Ssengooba, Achilles Katamba, William Worodria, Moses L. Joloba, Harriet Mayanja-Kizza

**Affiliations:** 1 Makerere University College of Heath Sciences, Kampala, Uganda; 2 Infectious Diseases Institute, Makerere University College of Health Sciences, Kampala, Uganda; University of Cape Town, South Africa

## Abstract

**Background:**

Diagnosis of pleural tuberculosis (TB) using routinely available diagnostic methods is challenging due to the paucibacillary nature of the disease. Histopathology and pleural tissue TB culture involves an invasive procedure which requires expertise and appropriate equipment, both often unavailable in many health units. Xpert MTB/Rif test has been widely evaluated in sputum specimens but data on its performance in pleural TB is scarce. We evaluated the accuracy of Cepheid's Xpert MTB/Rif test on pleural fluid in the diagnosis of pleural TB in Uganda.

**Methods:**

Consenting adult patients with exudative pleural effusions underwent pleural biopsy and the tissue obtained subjected to Lowenstein-Jensen and mycobacterial growth indicator tube MTB cultures and histopathology. Pleural fluid for Xpert MTB/Rif testing was also collected. Data on socio-demographic characteristics, clinical symptoms, HIV status and CD4 count were also collected. Sensitivity, specificity, positive and negative predictive values of Xpert MTB/Rif test on pleural fluid in pleural TB diagnosis were calculated using pleural tissue MTB culture and/or histopathology as the reference standard.

**Results:**

Of the 116 participants [female 50%, mean age 34 (SD ±13], 87/116 (75%) had pleural TB confirmed on pleural tissue culture and/or histopathology. The Xpert MTB/Rif test identified 25 (28.7%) of the 87 confirmed pleural TB cases. The sensitivity and specificity of Xpert MTB/Rif test were 28.7% and 96.6% respectively while the positive and negative predictive values were 96.1% and 31.1% respectively.

**Conclusion:**

Xpert MTB/Rif test on pleural fluid does not accurately diagnose pleural TB and therefore cannot be used as an initial evaluation test in patients with suspected pleural TB. New, rapid and accurate tests for the diagnosis of pleural TB are still warranted.

## Introduction

Tuberculosis (TB) is an important public health disease with a third of the world infected, of which 90% of these infections are in developing countries [1], [2]. TB/HIV co-infection has increased the incidence of extra pulmonary TB (EPTB), which is often fatal yet difficult to make a definite diagnosis [2], [3]. Pleural TB is the second commonest form of EPTB after TB adenitis [4] and its incidence has doubled in the last 20 years due to the HIV pandemic [4], [5]. TB pleural effusion occurs in approximately 5% of patients with *Mycobacterium tuberculosis* (MTB) infection [6]. Pleural TB accounted for 82% of all pleural effusions in a Rwanda study [7] and 91% of exudative pleural effusions in a Uganda study [5]. Accurate diagnosis and early treatment of TB has the potential to reduce pleural TB associated morbidity and mortality. However, diagnosis of pleural TB using conventional diagnostic methods is challenging [4], [8], [9]. Direct smear microscopy and solid MTB culture on pleural fluid have sensitivity less than 35% [8]–[10] although a slightly higher sensitivity up to 60% has been reported when liquid MTB culture media are used [11]. Pleural tissue MTB culture or histopathology, the gold standard for diagnosis, has varying sensitivity ranging from 40%–80% for pleural tissue MTB culture and 50%–97% for histopathology [12]–[15]. In addition, the pleural biopsy is an invasive procedure that requires skill and expertise as well as appropriate equipment, which are often unavailable in many health centers. The tissue MTB culture takes up to 8 weeks for LJ and as long as 6 weeks for liquid cultures to get final results [14], [15], and therefore may not be practically useful for rapid diagnosis of pleural TB where treatment has to be initiated as soon as possible to reduce associated morbidity and mortality. The newer serological tests like Interferon gamma release assays do not distinguish latent from active TB infection [9], [16]–[18].

Newer, more rapid tests such as nucleic-acid-amplification tests would provide more timely and accurate diagnosis of pleural TB and contribute to early initiation of TB treatment. Xpert MTB/RIF test (GeneXpert) is an integrated fully automated specimen processing real-time nucleic-acid-amplification test that detects MTB and rifampicin resistance within 2 hours [19]–[22]. Xpert MTB/Rif test is a promising innovation owing to its high sensitivity, specificity and rapid turnaround time [21]–[23]. The excellent performance of Xpert MTB/Rif test has mainly been demonstrated in sputum specimens but there is scarcity of data on its performance in EPTB specimens [24]–[29] including pleural TB diagnosis. The few available accuracy studies on Xpert MTB/Rif test on pleural fluid are limited by small sample sizes and have shown significantly low sensitivity [28]–[30]. In a recent study [30] the sensitivity and specificity of Xpert MTB/Rif on pleural fluid for pleura TB diagnosis was 15% and 100% respectively and in an earlier study [28] the sensitivity and specificity were 25% and 100% respectively. However, in both studies, the accuracy values were calculated among a relatively small number of pleural TB cases, (i.e. 33 and 20 pleural TB cases in the above two studies respectively). A South Africa study [29] which included 40 pleural TB cases also found low sensitivity (22.5%) of Xpert MTB/Rif test in the diagnosis of pleural fluid and the sensitivity did not improve even after pleural fluid was centrifuged.

In this study, we determined the accuracy of Xpert MTB/Rif test on pleural fluid for the diagnosis of pleural TB among adult patients in a high prevalence HIV/TB setting using pleural tissue TB culture and/or tissue histopathology as the reference standard. The aim was to provide additional information on the possible utility of Xpert MTB/Rif test as an option for accurate and timely diagnosis of pleural TB in a resource-limited setting.

### Ethics statement

The study was approved by the Makerere University School of Medicine research and ethics committee and the Mulago hospital ethics committee. Participants provided written informed consents before any study procedures were done. Results of pleural fluid analysis, pleural tissue histopathology and tissue MTB cultures were availed to the attending clinician to help in patient management.

## Methods

### Design and setting

This diagnostic study was carried out among inpatients and outpatients of Mulago National Referral Hospital, Kampala, Uganda. The enrolment period was April to December 2012.

### Study population

The target population was adult patients suspected of having pleural TB. Inclusion criteria were; i) age ≥18 years; ii) suspected pleural TB based on- clinical signs and symptoms, radiological evidence of a pleural effusion which was considered large enough for a pleural biopsy (>25% of a hemi-thorax) and exudative pleural effusion as defined by Light's criteria [31]; and iii) informed consent. Exclusion criteria were; having taken anti-TB treatment for more than two days within two months prior to enrollment, contraindications to pleural biopsy procedure and any other reason (by patient or researcher) that made it impossible for the participant to undergo a pleural biopsy procedure. Eligible participants were consecutively enrolled until the required sample size was attained.

### Screening and recruitment of participants

At screening, consenting patients who were clinically and radiologically suspected to have pleural TB underwent a thoracocentesis during which pleural fluid for analysis was obtained. Participants who had pleural fluid analysis indicative of an exudative pleural effusion using Light's criteria [31] were enrolled into the study. In summary, a participant was considered to have an exudative pleural effusion if pleural fluid Lactate Dehydrogenase (LDH) was greater than two thirds times the normal upper limit for serum LDH, which for this study was taken as 200 IU/L [32]. At enrollment, participants were interviewed for medical and demographic information and underwent a study specific physical examination. A pleural biopsy procedure to obtain pleural tissue for MTB culture and histopathology was performed under aseptic conditions. A minimum of four pleural tissue biopsy specimens were required, two of which were for MTB culture and the other two for histopathology. In situations where the tissue specimens were inadequate for both MTB cultures and histopathology, the available specimens were subjected to only tissue MTB culture. Prior to the pleural biopsy procedure, 5 to 10 ml of pleural fluid was collected for Xpert MTB/Rif testing. Blood for HIV testing for those of unknown HIV sero-status and CD4 cell count was also obtained.

### Laboratory Procedures

#### Xpert MTB/Rif testing

After collection, pleural fluid specimens were kept in a cool box until they were transferred to a refrigerator upon receipt in the Mycobacteriology laboratory (BSL-3) of Makerere University located within Mulago Hospital complex. Prior to Xpert MTB/Rif testing, the 5 to 10 ml of pleural fluid was centrifuged and supernatant poured off. The residue was then mixed with sample reagent for liquefaction and inactivation in a ratio of 2:1 before being transferred into a pre-labeled cartridge, which was then inserted into an Xpert MTB/Rif machine. After 2 hours of an automated cycle, a print out of results was obtained. Xpert MTB/Rif test was considered positive if MTB was detected. Repeat Xpert MTB/Rif tests were run for results that were indeterminate.

#### Pleural tissue MTB culture and histopathology

After the pleural biopsy procedure, two of the tissue specimens that were placed in a sterile bottle containing normal saline solution were sent to the Mycobacteriology laboratory (BSL-3) of Makerere University. Upon receipt in the laboratory, the tissue specimens were crushed in sterile normal saline solution to make a suspension using a mortar and pestle of which 1 ml was used to inoculate MGIT culture using MGIT 960 system (Becton and Dickinson, Franklin Lakes, NJ USA) and 0.5 ml to inoculate LJ culture media. Cultures were incubated at 37°C for up to six weeks for MGIT and eight weeks for LJ method. The results were recorded and interpreted according to standards [33], [34].

The other two tissue specimens that were also placed in a sterile bottle containing 10% formalin solution were sent to the Pathology laboratory of Makerere University for histopathology. Upon receipt in the laboratory, the tissue specimens were cut into 3-5µ slices and stained using Hematoxylin and Eosin staining technique in which the tissue slices were initially stained with Hematoxylin for 5–15 minutes, washed in water, dried in alcohol and then counter stained in Eosin. Presence of a necrotizing granuloma was indicative of TB.

Pleural fluid chemistry composition assessment to determine presence of an exudative pleural effusion was performed at the Mulago Hospital clinical chemistry laboratory.

HIV antibody testing using ELISA and CD4 cell enumerations were performed at a certified laboratory at the Infectious Diseases Institute, Makerere College of Health Sciences located within Mulago Hospital Complex.

All study TB laboratory results were made available to the attending clinicians. Discharged participants were contacted by telephone to avail TB results and those whose TB tests were positive were requested to return for TB treatment. MTB positive patients by Xpert MTB/Rif on pleural fluid, tissue MTB culture and/or histopathology were immediately initiated on TB treatment by the attending clinician according to the guidelines from the Uganda Ministry of Health TB and Leprosy program [35]. Participants were confirmed as pleural TB cases after isolation of MTB on pleural tissue MTB culture and/or if there was presence of histopathology description of TB (i.e. necrotizing granuloma).

### Statistical analysis

Descriptive statistics were generated to describe the baseline characteristic of the study population. Continuous variables were summarized into means and standard deviations (SD), medians and inter-quartile range (IQR), and categorical variables in frequencies and percentages. Exploratory inferential statistics were generated to determine statistical difference between the groups using Chi-square test for categorical variables and Student's T-test and Wilcoxon rank-sum test for continuous normally distributed and non-normally distributed variables respectively. Fisher's exact test was used for variables less than five in a given cell. To determine the accuracy of Xpert MTB/Rif test on pleural fluid in the diagnosis of pleural TB, sensitivity, specificity, positive and negative predictive values with confidence intervals were calculated using pleural tissue MTB culture and/or histopathology as the reference standard. A p-value of ≤0.05 was considered statistically significant. All data were analyzed using STATA version 12.0 (StataCorp, 4905 Lakeway Drive College Station, Texas USA).

## Results

### Participants' baseline characteristics

**Figure 1:**
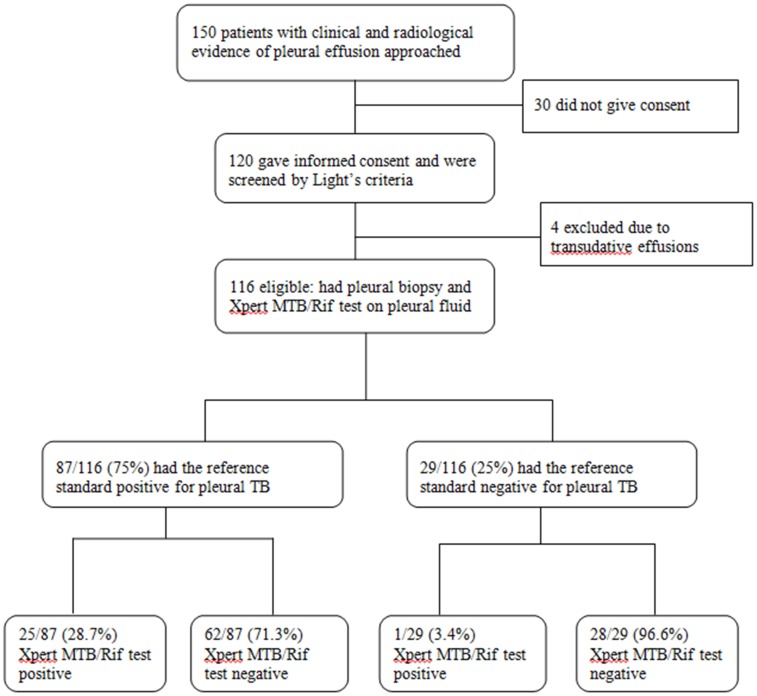
Patient enrollment flow diagram showing the number of patients enrolled and analyzed.

Of the 150 adult patients with clinical and radiologic evidence of pleural effusion approached, 120 gave written informed consent. Of these, 116/120 (96.7%) had exudative pleural effusion based on Light's criteria and were therefore eligible for the study. The remaining four had results suggestive of transudative pleural effusion and were excluded (**Figure 1**). Characteristics of the enrolled 116 participants are summarized in **Table 1**. The mean (±SD) age was 34±13 years, 57% males, majority (83.6%) were non-smokers; 52 (44.8%) of the 116 participants were HIV-infected with median CD4 (IQR) 154 (100–236) cells/mm^3^.

**Table 1:** Comparing characteristics of study participants with exudative pleural effusions with and without pleural TB.

Characteristics	Overall (N = 116)	Pleural (N = 87)	No pleural TB (N = 29)	P value
Mean age (±SD) years	34 (±13)	32 (±11)	41 (±15)	<0.001
Females, n (%)	50 (43.1)	39 (44.8)	11 (37.9)	0.516
Cough for ≥2 weeks, n (%)	95 (81.9)	69 (90.8)	26 (89.6)	0.358
Weight loss, n (%)	93 (80.2)	70 (80.5)	23 (79.3)	0.893
Fever	106 (91.4)	79 (90.8)	27 (93.1)	0.826
Excessive night sweats	83 (71.6)	62 (71.3)	21 (72.4)	0.905
Pleuritic chest pain, n (%)	113 (97.4)	85 (97.7)	20 (68.9)	0.736
Smoking, n (%)	19 (16.4)	10 (11.5)	9 (31)	0.014
Alcohol	41 (35.3)	29 (33.3)	12 (41.3)	0.432
Mean weight (±SD) KG	57.1 (±7.3)	56.6 (±7.4)	58.6 (±7.2)	0.218
Median KPS (IQR)	80 (70–80)	80 (70–80)	80 (70–80)	0.655
Temperature >37.4°C	34 (29.3)	30 (34.5)	4 (13.8)	0.034
HIV positive, n (%)	52 (44.8)	41 (47.1)	11 (37.9)	0.389
**Median CD4 (IQR) (cells/mm^3^)	154(100–236)	112 (90–210)	136(100–206)	0.132
PF LDH >1000 (IU/L), n(%)	11(9.5)	7 (8.0)	4 (13.8)	0.36

**CD4 cell counts only performed for HIV positive participants.

**Abbreviations**: SD, Standard deviations; KPS, Karnofsky performance scale; IQR, Inter-quartile range; PF, Pleural fluid; LDH, Lactate Dehydrogenase.

The main presenting respiratory symptoms were cough (95/116, 81.9%) and pleuritic chest pain (113/116, 97.4%). Constitutional symptoms including fever (106/116, 91.4%), excessive night sweats (83/116, 71.6%) and weight loss of >10% in 4 weeks (93/116, 80.2%) were also common among the participants.

### Tissue MTB culture and histopathology results

Of the 116 participants, 104 (90%) had both tissue MTB culture and histopathology results available as reference tests, the other 12 had only tissue MTB culture available (histopathology was not performed due to inadequate specimens). Presence of a positive result on any or both of the reference standard tests was considered as confirmed pleural TB.

Pleural TB was confirmed in 87/116 (75%) of the participants, of which 81.6% (71/87) had pleural TB confirmed on both tissue MTB culture and histopathology, the remaining 18.4% (16/87) had the diagnosis confirmed on either one of the reference standard tests. Of the 29 participants who did not have a confirmed diagnosis of pleural TB by the reference tests, 18 had other non-TB histological diagnoses which included non-specified chronic inflammation (8), Kaposi sarcoma (3), mesothelioma (3) and other unspecified malignancies which required further cytological analysis (4). Eleven biopsy tissue specimens were reported as non-pleural (muscle or fibrous tissue) and were therefore non-diagnostic. Follow up information on the diagnosis of these eleven patients was not available.

Participants with pleura TB were younger when compared to those without pleural TB [mean (±SD) age 32±11 years versus 41±15 years respectively, p<0.001] and were more likely to have a high temperature >37.4°C (34.5% versus 13.8% respectively, p = 0.034) (**Table 1**). There were more participants without pleural TB who smoked cigarettes when compared to participants with pleural TB [31% (9/29) versus 11.5%, (10/87), p = 0.014 respectively].

The proportion of participants who were HIV-infected was not significantly different between the participants with and without pleural TB [47% (41/87) among pleural TB participants versus 38% (11/29) among non-pleural TB, p = 0.389].

### Performance of Xpert MTB/Rif test on pleural fluid for diagnosis of pleural TB

The accuracy of the Xpert MTB/Rif test on pleural fluid for the diagnosis of pleural TB using pleural tissue MTB culture and/or histopathology as the reference standard was determined (**Table 2**). Xpert MTB/Rif test was positive in 25 of the 87 pleural TB confirmed participants and the sensitivity and specificity were 28.7% (25/87) and 96.6% (28/29) respectively. The positive and negative predictive values were 96.1% (25/26) and 31.1% (28/90) respectively. One HIV-infected male participant (CD4 cell count  = 114 cells/mm^3^) had a positive Xpert MTB/Rif test but both reference standard tests were negative. This participant improved on empirical anti-TB medication. None of the Xpert MTB/Rif positive participants had rifampicin resistance reported. Accuracy values for Xpert MTB/Rif test on pleural fluid for the diagnosis of pleural TB when participants were stratified by HIV status are shown in **Table 2**. The sensitivity and specificity according to HIV status were 36. 6% and 90.9% respectively among the HIV-infected participants and 21.7% and 100% respectively among the HIV-uninfected. LDH >1000 IU/L was more common among participants with a positive Xpert MTB/Rif test when compared to the Xpert MTB/Rif test negative participants (data not shown). There was no significant difference in HIV positivity between the Xpert MTB/Rif test positive and negative pleural TB participants (60% versus 42% respectively, p = 0.127) (data not shown).

**Table 2:** Accuracy of Xpert MTB/Rif test on pleural fluid in pleural TB diagnosis using pleural tissue MTB culture and/or histopathology as reference standard, stratified by HIV status.

	Overall accuracy, N = 116, Estimated (95% CI)	HIV-uninfected, N = 64, Estimate (95% CI)	HIV-infected, N = 52, Estimate (95% CI)
Sensitivity	28.7% (19.5%, 39.4%)	21.7% (10.9%, 36.4%)	36.6% (22.1%, 53.1%)
Specificity	96.6% (82.2%, 99.9%)	100.0% (81.5%, 100.0)%	90.9% (58.7%, 99.8%)
PPV	96.1% (80.4%, 99.9%)	100.0% (69.1%, 100.0%)	93.8% (69.8%, 99.8%)
NPV	31.1% (21.8%, 41.7%)	33.3% (21.1%, 47.5%)	27.8% (14.2%, 45.2%)

**Abbreviations**: PPV, positive predictive value; NPV, negative predictive value; CI, confidence intervals; N, number of participants.

## Discussion

In this high prevalence TB setting, pleural TB occurred in two thirds of patients presenting with exudative pleural effusions. Xpert MTB/Rif test on pleural effusion showed high specificity but low sensitivity in the diagnosis of pleural TB when compared to pleural tissue MTB culture and/or histopathology as reference standard.

The high proportion of pleural TB cases among patients presenting with exudative pleural effusion obtained in our study is similar to an earlier report from the same setting [5] where 91% of patients with exudative pleural effusion had pleural TB. TB has also been reported as the commonest cause of pleural effusions in other settings [4], [7], [8], [14]. This finding confirms TB as the commonest cause of exudative pleural effusions in most settings especially where the prevalence of TB is high.

Similar to earlier reports [25]–[30], we found a low sensitivity and a high specificity of Xpert MTB/Rif test on pleural fluid in the diagnosis of pleural TB. Specifically, the earlier reports which are mainly from smaller studies [26]–[30] reported low sensitivity of Xpert MTB/Rif test on pleural fluid ranging between 15–33% but high specificity up to 100%. The low sensitivity of Xpert MTB/Rif on pleural fluid could be explained by the low mycobacterial load in pleural fluid [8]. Also similar to these earlier studies [26]–[30], Xpert MTB/Rif test correctly identified 96.6% of non-pleural TB participants as no TB (high specificity) and pleural TB was confirmed present in 96.1% of Xpert MTB/Rif test positive patients (high positive predictive value). The high specificity and positive predictive value of Xpert MTB/Rif test on pleural effusion gives a clinician confidence to make a diagnosis of pleural TB when Xpert MTB/Rif test on pleural fluid is positive. However, our study shows that a negative Xpert MTB/Rif test does not completely exclude the diagnosis of pleural TB given the fact that the test was unable to identify 71.3% of confirmed pleural TB cases (low sensitivity) and when Xpert MTB/Rif test was negative, 69% of patients still had pleural TB. The findings imply that clinical decision in combination with routine pleural fluid analysis is still crucial in the diagnosis of pleural TB in clinically and radiologically suspected patients.

The performance values of Xpert MTB/Rif test on pleural fluid remained almost similar when patients were stratified by HIV status and this is likely due to the low mycobacterial load seen in pleural TB. We documented one participant who had a positive Xpert MTB/Rif test but both reference standard results were negative for pleural TB. This HIV positive participant with low CD4 counts could have been missed by both tissue MTB culture and histopathology due to low mycobactereology load and inability to form granulomas respectively, as commonly found in HIV/TB co infection [36].

In our study, a positive pleural fluid Xpert MTB/Rif test was more likely in patients who had pleural fluid LDH levels >1000 IU/L. This suggests that in the absence of pleural biopsy, this expensive but rapid new test could be used in patients whose pleural fluid analysis has shown high levels of LDH >1000 IU/L.

The study had a few limitations. We did not perform some of the tests that are known to be indicative of pleural TB including markers such as adenosine deaminase and Interferon gamma assays because these are not readily available for diagnosis in this setting. We were also unable to perform TB culture on pleural fluid and therefore unable to compare the performance of Xpert MTB/Rif test on pleural fluid to pleural fluid TB culture in the diagnosis of pleural TB.

Our study has important strengths. In order to classify the patients correctly as having pleural TB, we performed both pleural tissue MTB culture (on both solid and liquid culture) and histopathology that were used as the reference standard, making our findings reliable and accurate. Our study is one of the largest clinical studies evaluating performance of Xpert MTB/Rif test on pleural fluid for the diagnosis of pleural TB in a high prevalence TB/HIV resource- limited setting in which both inpatients and outpatients participants were consecutively recruited and underwent protocol-specified procedures. Most of the other reported studies had small sample sizes, had designs that made them prone to selection and information bias and majority included either only HIV infected or HIV negative participants.

In conclusion, Xpert MTB/Rif test on pleural fluid does not correctly diagnose pleural TB in the majority of pleural tissue culture or histopathology confirmed pleural TB cases and therefore may not be implemented for use in the evaluation of patients with pleural effusions to diagnose pleural TB. New, rapid and accurate tests for the diagnosis of pleural TB are still warranted. We recommend further evaluation of Xpert MTB/Rif test for pleural TB diagnosis in HIV-infected patients at various levels of immunosuppression.
